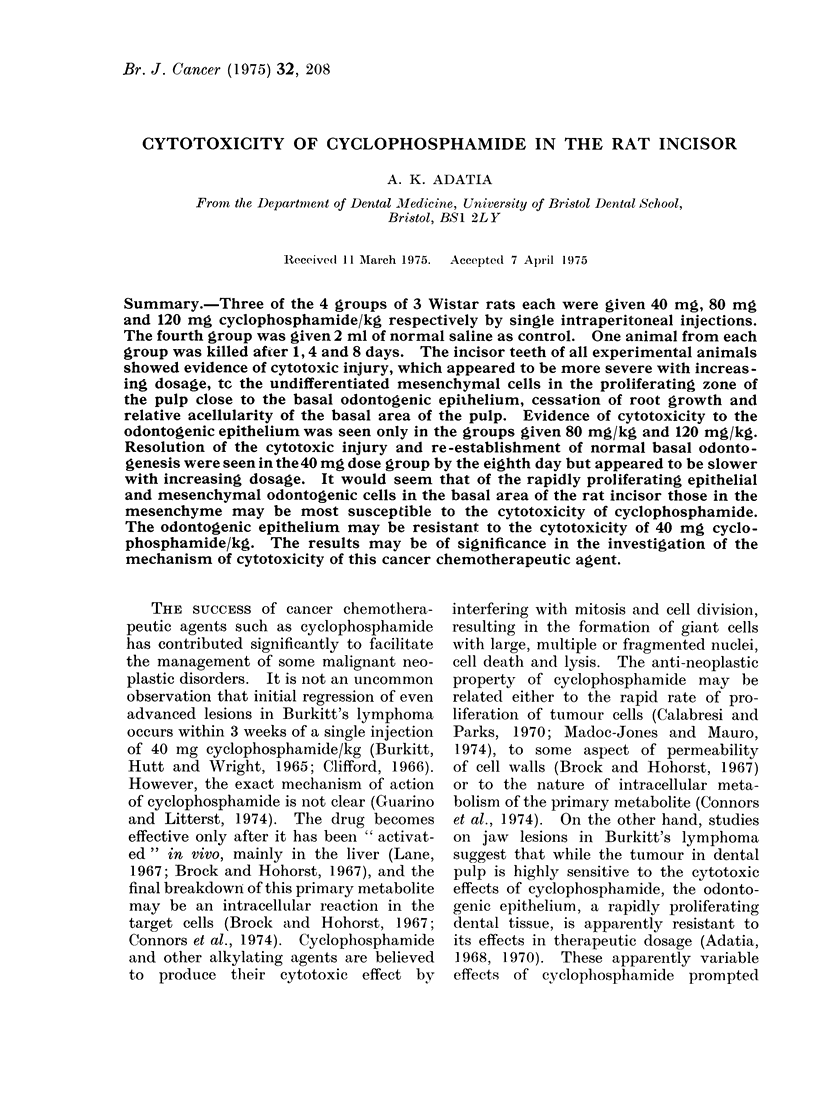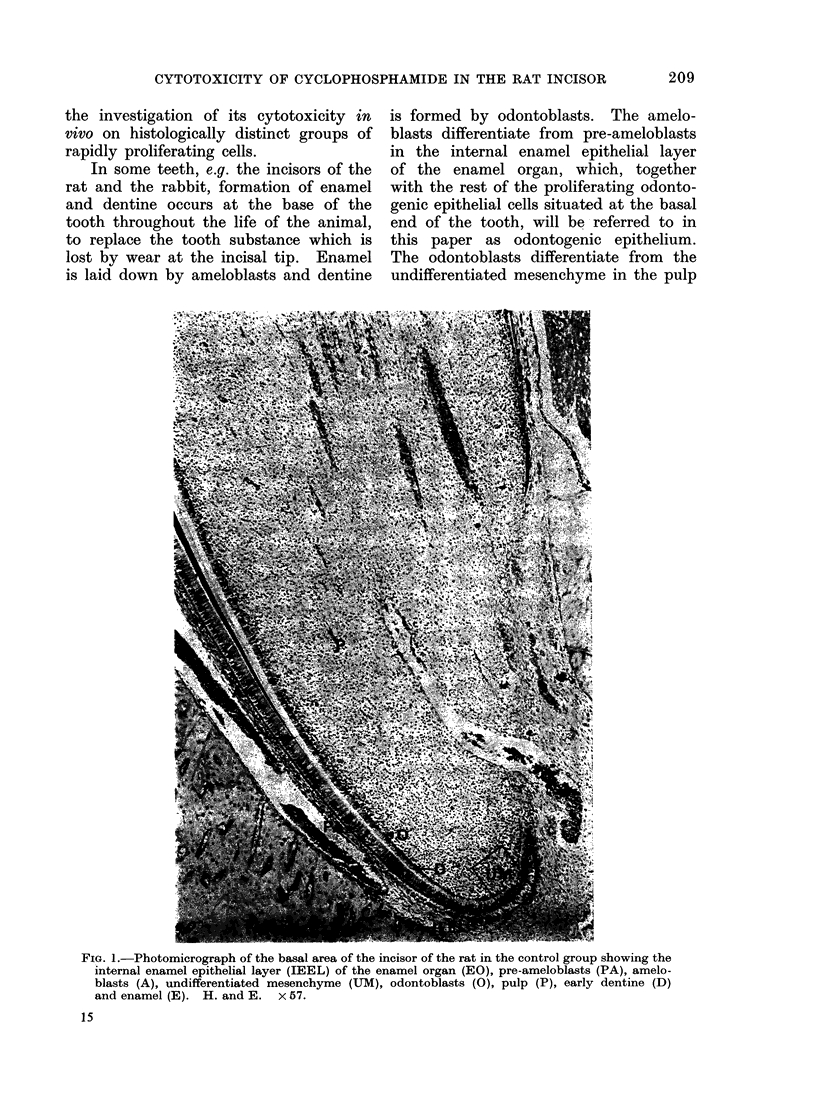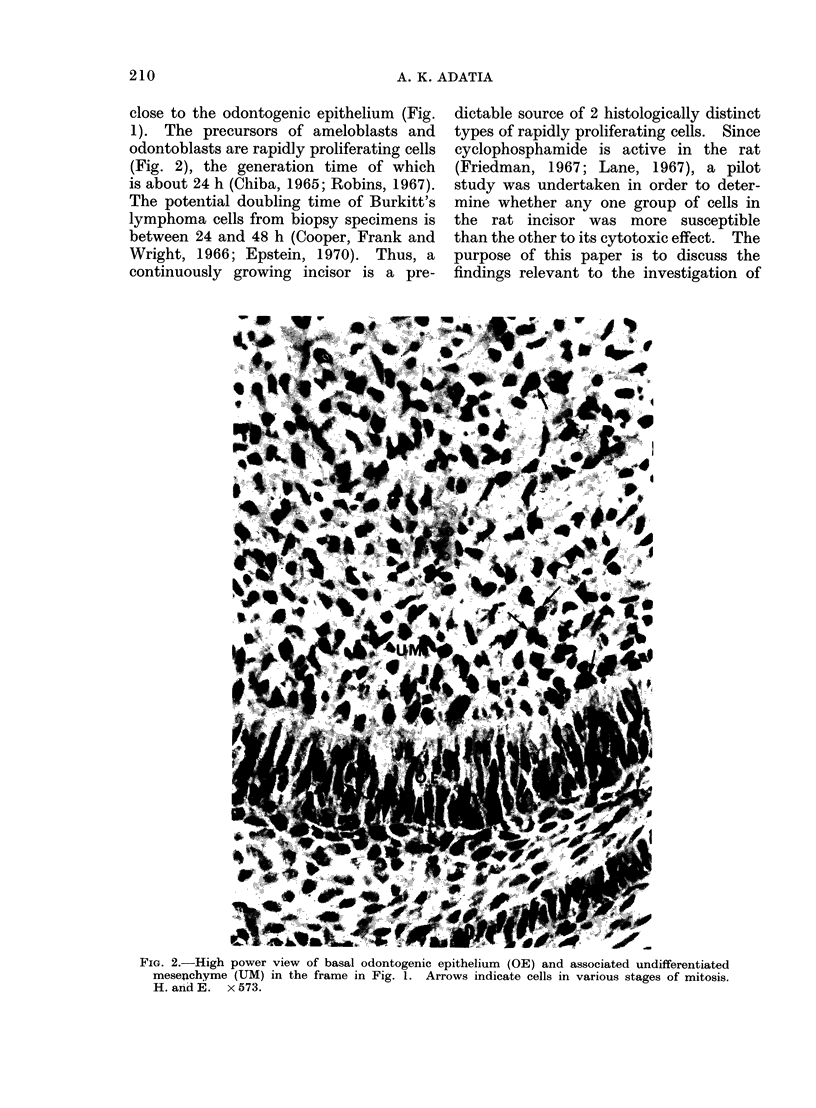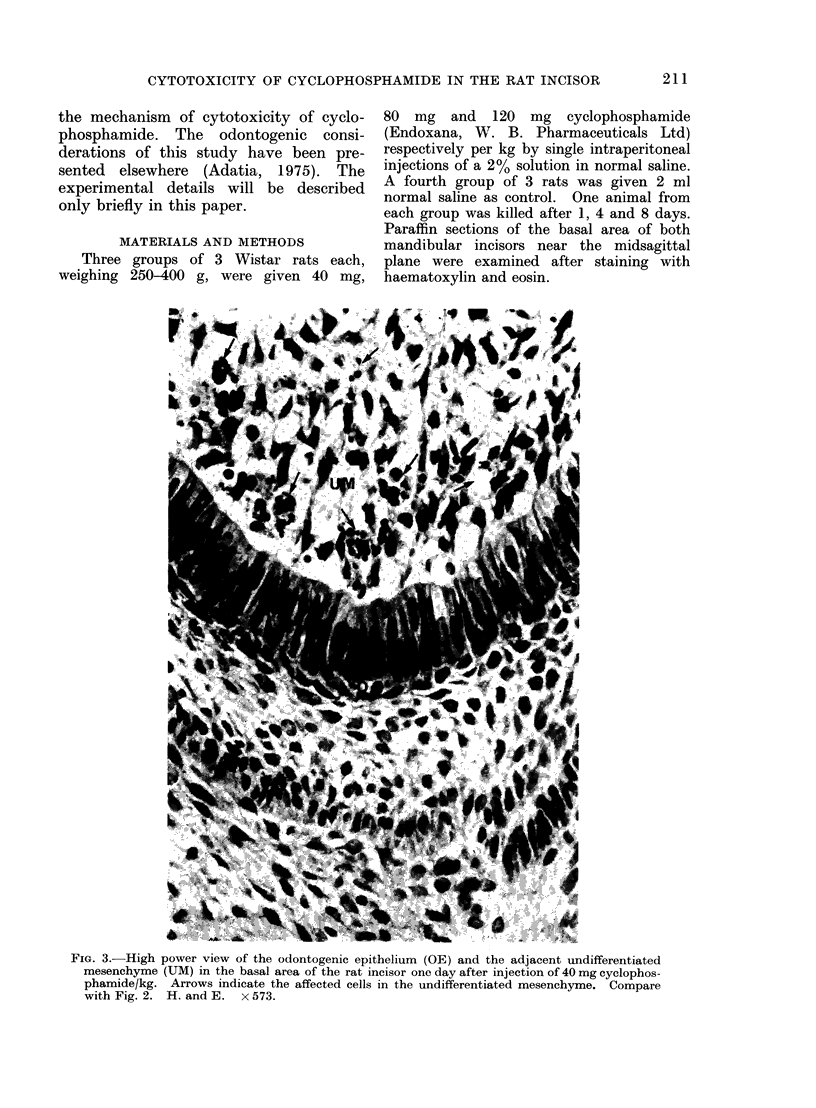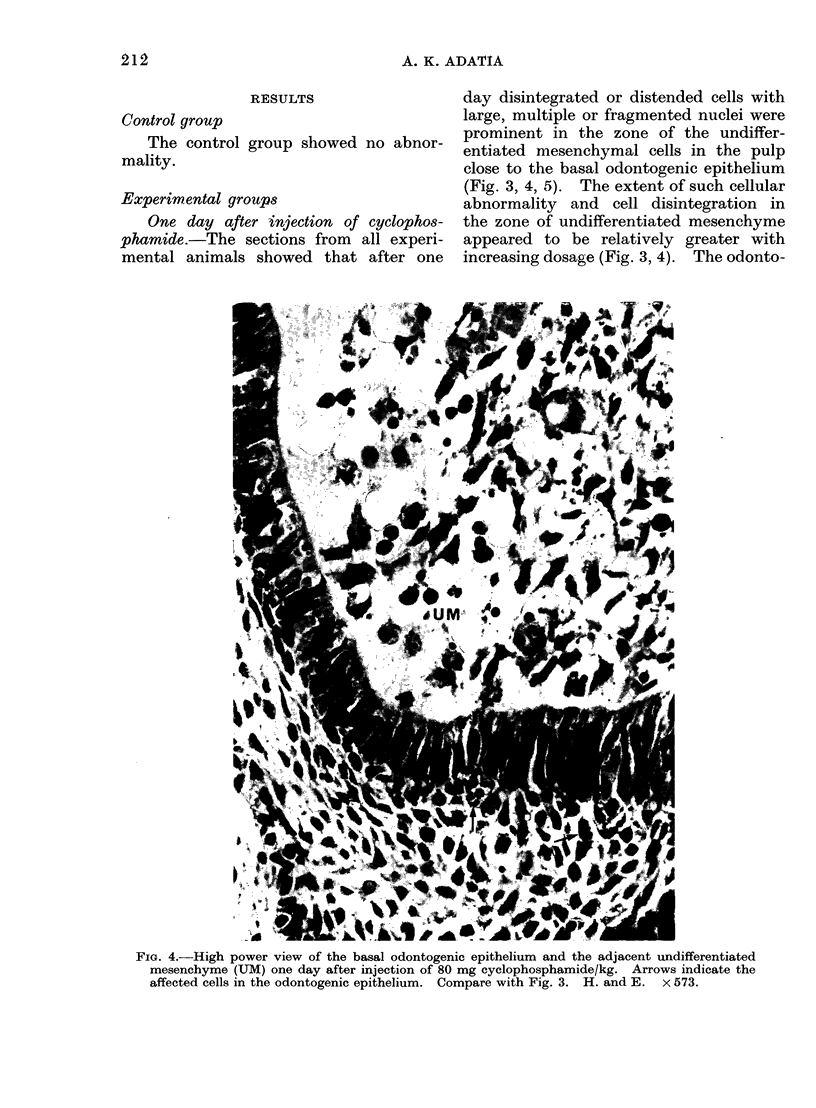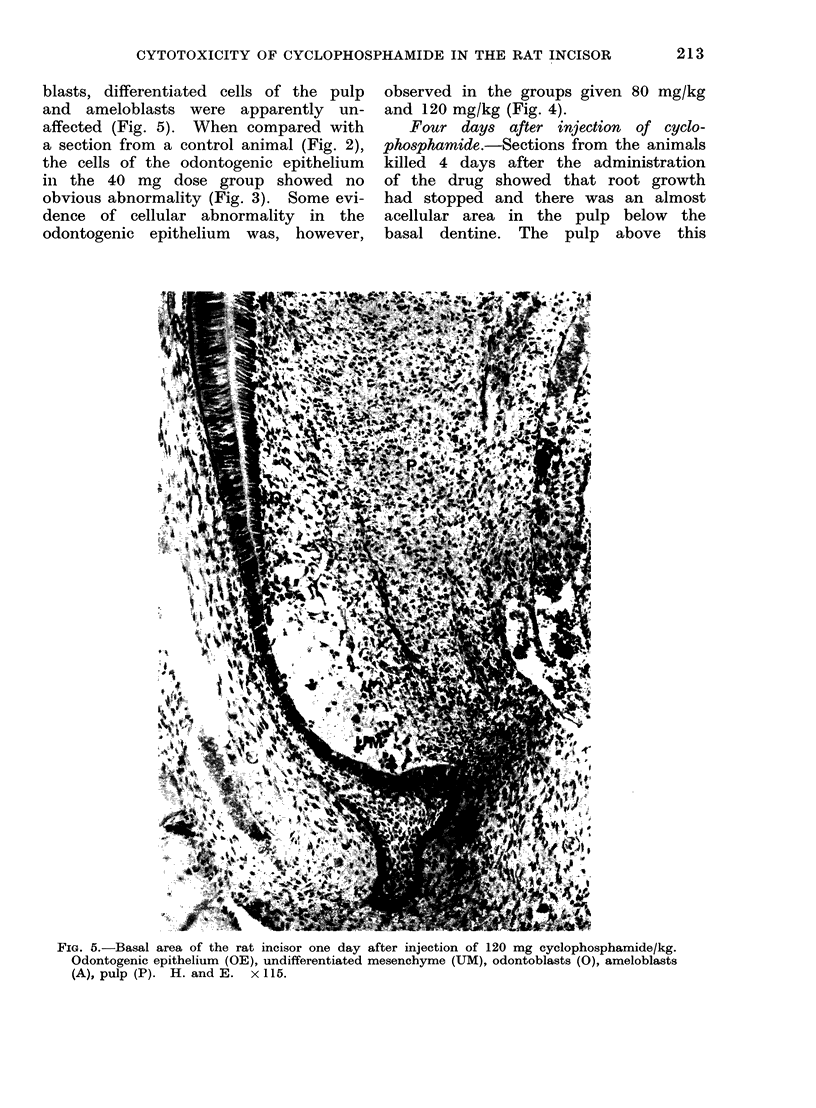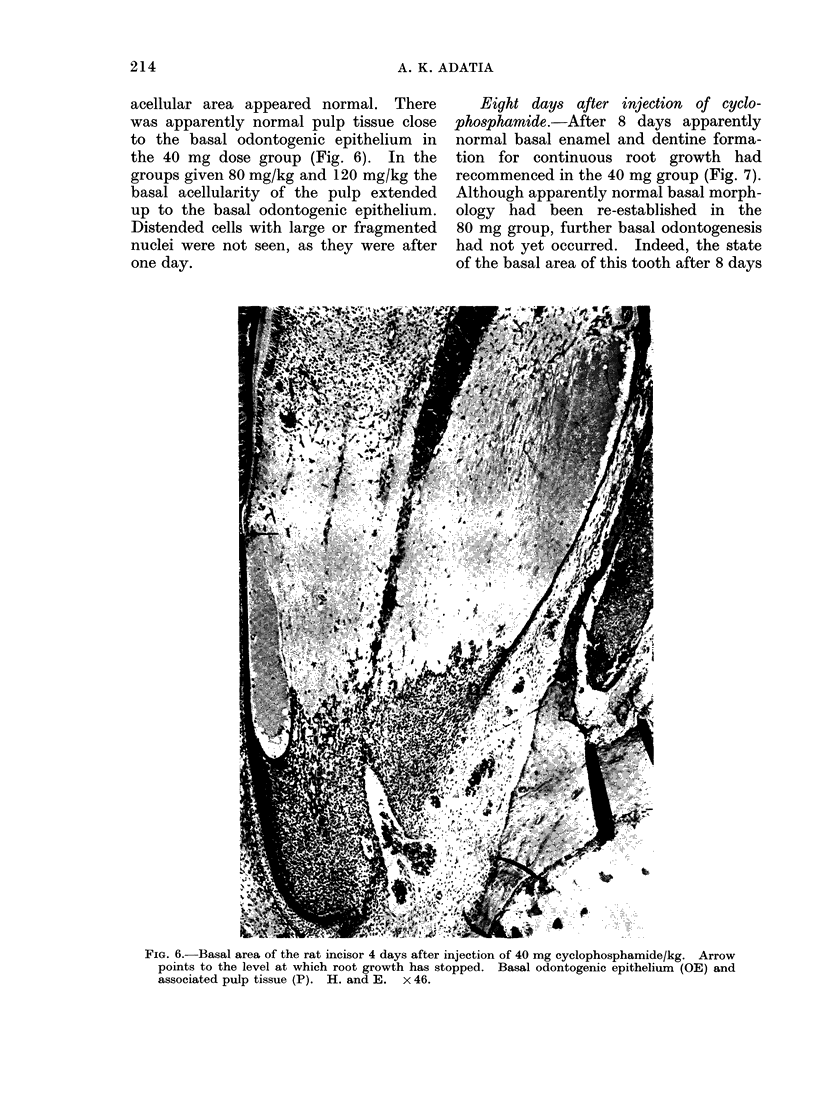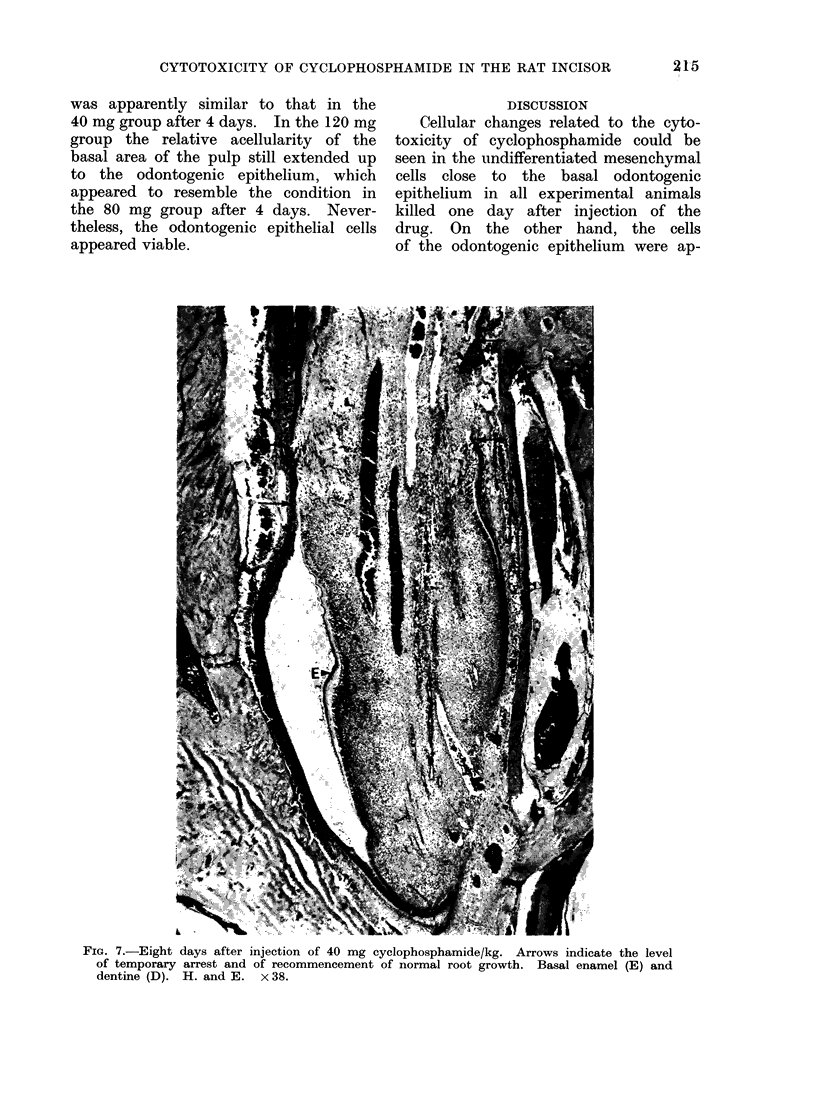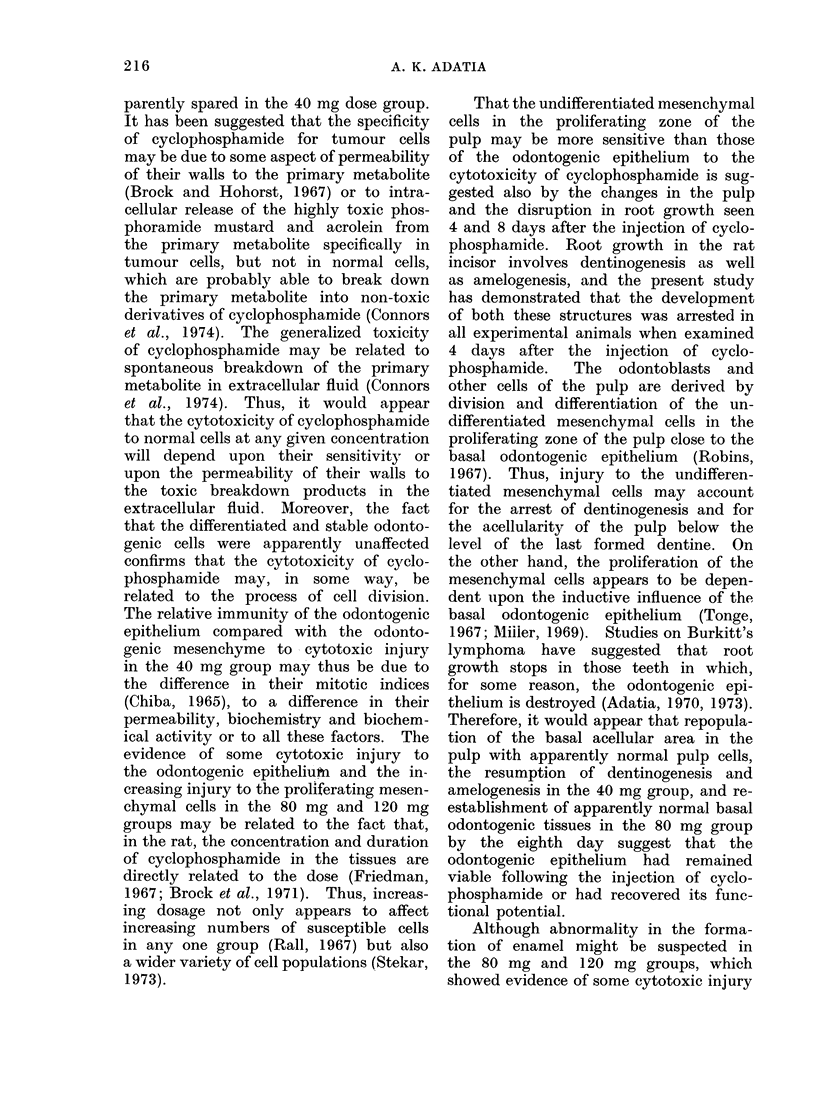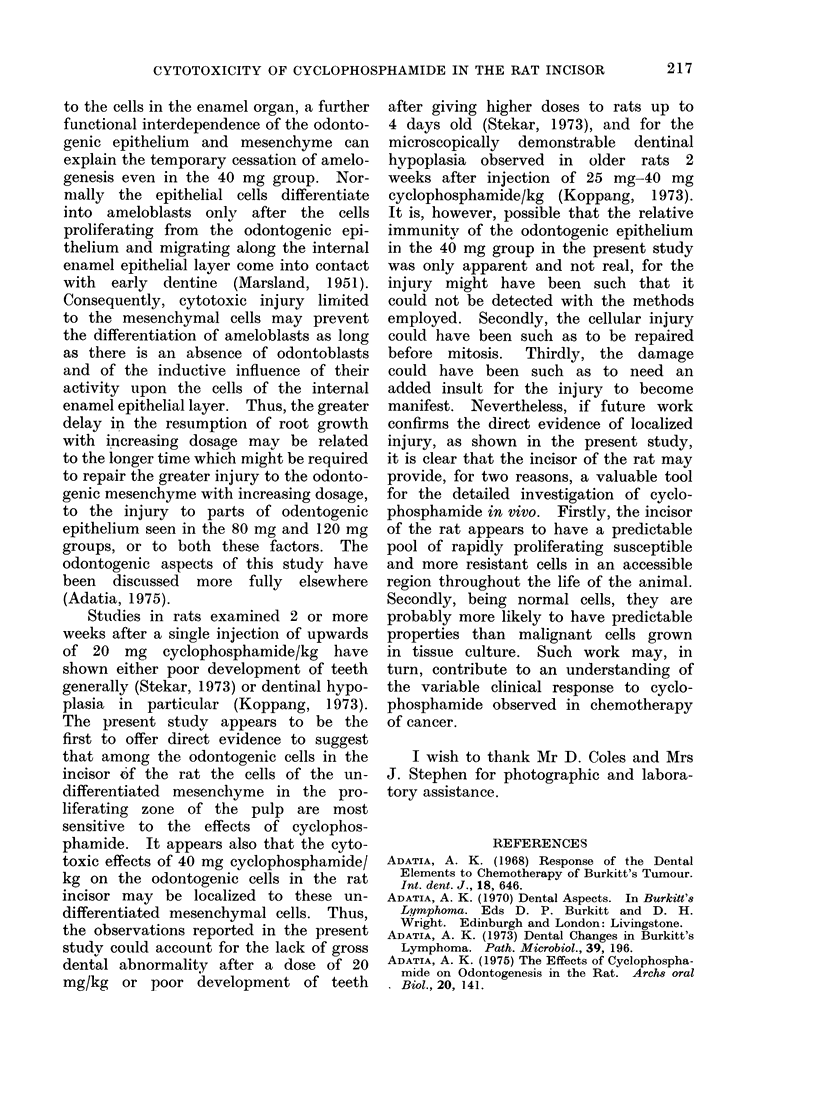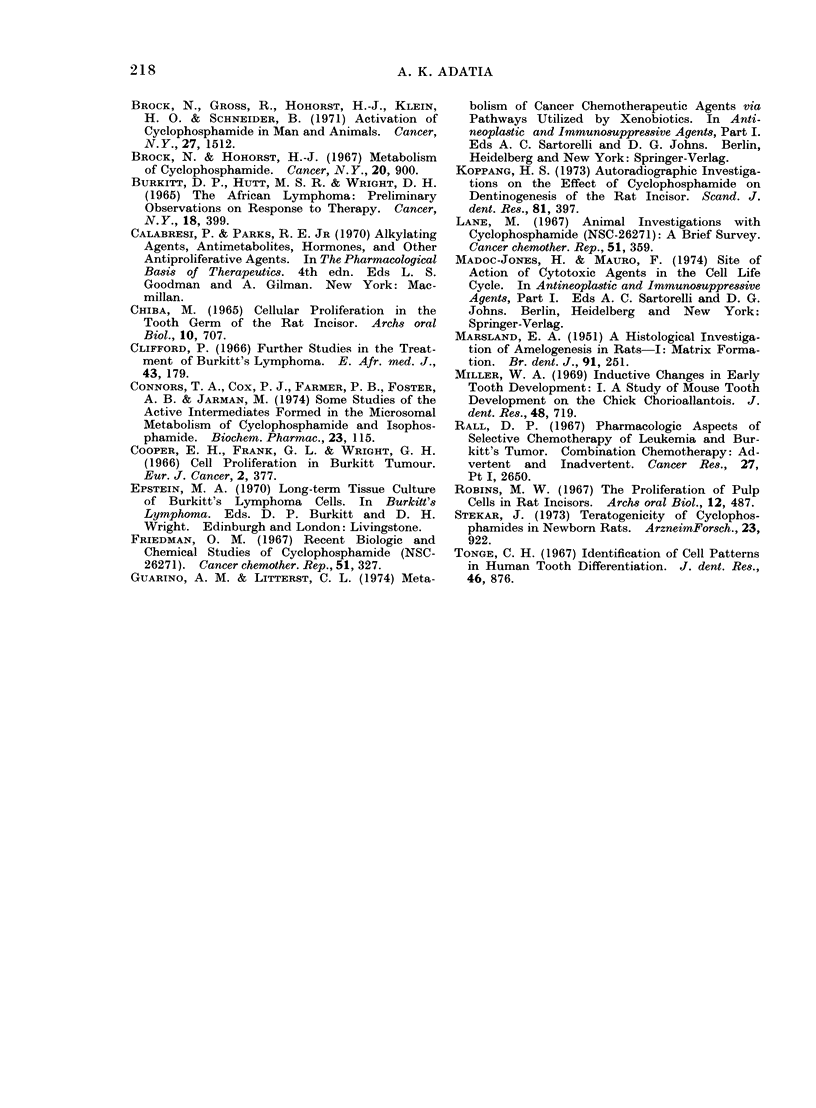# Cytotoxicity of cyclophosphamide in the rat incisor.

**DOI:** 10.1038/bjc.1975.151

**Published:** 1975-08

**Authors:** A. K. Adatia

## Abstract

**Images:**


Br. J. Cancer (1975) 32, 208

### CYTOTOXICITY OF CYCLOPHOSPHAMIDE IN THE RAT INCISOR

A. K. ADATIA

*From the Department of Dental Medicine, University of Bristol Dental School, Bristol, BS1 2LY*

Received 11 March 1975. Accepted 7 April 1975

**Summary.—Three of the 4 groups of 3 Wistar rats each were given 40 mg, 80 mg and 120 mg cyclophosphamide/kg respectively by single intraperitoneal injections. The fourth group was given 2 ml of normal saline as control. One animal from each group was killed after 1, 4 and 8 days. The incisor teeth of all experimental animals showed evidence of cytotoxic injury, which appeared to be more severe with increasing dosage, tc the undifferentiated mesenchymal cells in the proliferating zone of the pulp close to the basal odontogenic epithelium, cessation of root growth and relative acellularity of the basal area of the pulp. Evidence of cytotoxicity to the odontogenic epithelium was seen only in the groups given 80 mg/kg and 120 mg/kg. Resolution of the cytotoxic injury and re-establishment of normal basal odontogenesis were seen in the 40 mg dose group by the eighth day but appeared to be slower with increasing dosage. It would seem that of the rapidly proliferating epithelial and mesenchymal odontogenic cells in the basal area of the rat incisor those in the mesenchyme may be most susceptible to the cytotoxicity of cyclophosphamide. The odontogenic epithelium may be resistant to the cytotoxicity of 40 mg cyclophosphamide/kg. The results may be of significance in the investigation of the mechanism of cytotoxicity of this cancer chemotherapeutic agent.**

THE SUCCESS of cancer chemotherapeutic agents such as cyclophosphamide has contributed significantly to facilitate the management of some malignant neoplastic disorders. It is not an uncommon observation that initial regression of even advanced lesions in Burkitt's lymphoma occurs within 3 weeks of a single injection of 40 mg cyclophosphamide/kg (Burkitt, Hutt and Wright, 1965; Clifford, 1966). However, the exact mechanism of action of cyclophosphamide is not clear (Guarino and Litterst, 1974). The drug becomes effective only after it has been "activated" *in vivo*, mainly in the liver (Lane, 1967; Brock and Hohorst, 1967), and the final breakdown of this primary metabolite may be an intracellular reaction in the target cells (Brock and Hohorst, 1967; Connors *et al.*, 1974). Cyclophosphamide and other alkylating agents are believed to produce their cytotoxic effect by interfering with mitosis and cell division, resulting in the formation of giant cells with large, multiple or fragmented nuclei, cell death and lysis. The anti-neoplastic property of cyclophosphamide may be related either to the rapid rate of proliferation of tumour cells (Calabresi and Parks, 1970; Madoc-Jones and Mauro, 1974), to some aspect of permeability of cell walls (Brock and Hohorst, 1967) or to the nature of intracellular metabolism of the primary metabolite (Connors *et al.*, 1974). On the other hand, studies on jaw lesions in Burkitt's lymphoma suggest that while the tumour in dental pulp is highly sensitive to the cytotoxic effects of cyclophosphamide, the odontogenic epithelium, a rapidly proliferating dental tissue, is apparently resistant to its effects in therapeutic dosage (Adatia, 1968, 1970). These apparently variable effects of cyclophosphamide prompted the investigation of its cytotoxicity *in vivo* on histologically distinct groups of rapidly proliferating cells.

In some teeth, *e.g.* the incisors of the rat and the rabbit, formation of enamel and dentine occurs at the base of the tooth throughout the life of the animal, to replace the tooth substance which is lost by wear at the incisal tip. Enamel is laid down by ameloblasts and dentine is formed by odontoblasts. The ameloblasts differentiate from pre-ameloblasts in the internal enamel epithelial layer of the enamel organ, which, together with the rest of the proliferating odontogenic epithelial cells situated at the basal end of the tooth, will be referred to in this paper as odontogenic epithelium. The odontoblasts differentiate from the undifferentiated mesenchyme in the pulp

FIG. 1.—Photomicrograph of the basal area of the incisor of the rat in the control group showing the internal enamel epithelial layer (IEEL) of the enamel organ (EO), pre-ameloblasts (PA), ameloblasts (A), undifferentiated mesenchyme (UM), odontoblasts (O), pulp (P), early dentine (D) and enamel (E). H. and E. ×57.

close to the odontogenic epithelium (Fig. 1). The precursors of ameloblasts and odontoblasts are rapidly proliferating cells (Fig. 2), the generation time of which is about 24 h (Chiba, 1965; Robins, 1967). The potential doubling time of Burkitt's lymphoma cells from biopsy specimens is between 24 and 48 h (Cooper, Frank and Wright, 1966; Epstein, 1970). Thus, a continuously growing incisor is a predictable source of 2 histologically distinct types of rapidly proliferating cells. Since cyclophosphamide is active in the rat (Friedman, 1967; Lane, 1967), a pilot study was undertaken in order to determine whether any one group of cells in the rat incisor was more susceptible than the other to its cytotoxic effect. The purpose of this paper is to discuss the findings relevant to the investigation of

FIG. 2.—High power view of basal odontogenic epithelium (OE) and associated undifferentiated mesenchyme (UM) in the frame in Fig. 1. Arrows indicate cells in various stages of mitosis. H. and E. ×573.

the mechanism of cytotoxicity of cyclophosphamide. The odontogenic considerations of this study have been presented elsewhere (Adatia, 1975). The experimental details will be described only briefly in this paper.

### MATERIALS AND METHODS

Three groups of 3 Wistar rats each, weighing 250–400 g, were given 40 mg, 80 mg and 120 mg cyclophosphamide (Endoxana, W. B. Pharmaceuticals Ltd) respectively per kg by single intraperitoneal injections of a 2% solution in normal saline. A fourth group of 3 rats was given 2 ml normal saline as control. One animal from each group was killed after 1, 4 and 8 days. Paraffin sections of the basal area of both mandibular incisors near the midsagittal plane were examined after staining with haematoxylin and eosin.

FIG. 3.—High power view of the odontogenic epithelium (OE) and the adjacent undifferentiated mesenchyme (UM) in the basal area of the rat incisor one day after injection of 40 mg cyclophosphamide/kg. Arrows indicate the affected cells in the undifferentiated mesenchyme. Compare with Fig. 2. H. and E. ×573.

RESULTS

*Control group*

The control group showed no abnormality.

*Experimental groups*

*One day after injection of cyclophosphamide.*—The sections from all experimental animals showed that after one day disintegrated or distended cells with large, multiple or fragmented nuclei were prominent in the zone of the undifferentiated mesenchymal cells in the pulp close to the basal odontogenic epithelium (Fig. 3, 4, 5). The extent of such cellular abnormality and cell disintegration in the zone of undifferentiated mesenchyme appeared to be relatively greater with increasing dosage (Fig. 3, 4). The odonto-

FIG. 4.—High power view of the basal odontogenic epithelium and the adjacent undifferentiated mesenchyme (UM) one day after injection of 80 mg cyclophosphamide/kg. Arrows indicate the affected cells in the odontogenic epithelium. Compare with Fig. 3. H. and E. ×573.

blasts, differentiated cells of the pulp and ameloblasts were apparently unaffected (Fig. 5). When compared with a section from a control animal (Fig. 2), the cells of the odontogenic epithelium in the 40 mg dose group showed no obvious abnormality (Fig. 3). Some evidence of cellular abnormality in the odontogenic epithelium was, however, observed in the groups given 80 mg/kg and 120 mg/kg (Fig. 4).

*Four days after injection of cyclophosphamide.*—Sections from the animals killed 4 days after the administration of the drug showed that root growth had stopped and there was an almost acellular area in the pulp below the basal dentine. The pulp above this

FIG. 5.—Basal area of the rat incisor one day after injection of 120 mg cyclophosphamide/kg. Odontogenic epithelium (OE), undifferentiated mesenchyme (UM), odontoblasts (O), ameloblasts (A), pulp (P). H. and E. ×115.

acellular area appeared normal. There was apparently normal pulp tissue close to the basal odontogenic epithelium in the 40 mg dose group (Fig. 6). In the groups given 80 mg/kg and 120 mg/kg the basal acellularity of the pulp extended up to the basal odontogenic epithelium. Distended cells with large or fragmented nuclei were not seen, as they were after one day.

*Eight days after injection of cyclophosphamide.*—After 8 days apparently normal basal enamel and dentine formation for continuous root growth had recommenced in the 40 mg group (Fig. 7). Although apparently normal basal morphology had been re-established in the 80 mg group, further basal odontogenesis had not yet occurred. Indeed, the state of the basal area of this tooth after 8 days

FIG. 6.—Basal area of the rat incisor 4 days after injection of 40 mg cyclophosphamide/kg. Arrow points to the level at which root growth has stopped. Basal odontogenic epithelium (OE) and associated pulp tissue (P). H. and E. ×46.

was apparently similar to that in the 40 mg group after 4 days. In the 120 mg group the relative acellularity of the basal area of the pulp still extended up to the odontogenic epithelium, which appeared to resemble the condition in the 80 mg group after 4 days. Nevertheless, the odontogenic epithelial cells appeared viable.

### DISCUSSION

Cellular changes related to the cytotoxicity of cyclophosphamide could be seen in the undifferentiated mesenchymal cells close to the basal odontogenic epithelium in all experimental animals killed one day after injection of the drug. On the other hand, the cells of the odontogenic epithelium were ap-

FIG. 7.—Eight days after injection of 40 mg cyclophosphamide/kg. Arrows indicate the level of temporary arrest and of recommencement of normal root growth. Basal enamel (E) and dentine (D). H. and E. ×38.

parently spared in the 40 mg dose group. It has been suggested that the specificity of cyclophosphamide for tumour cells may be due to some aspect of permeability of their walls to the primary metabolite (Brock and Hohorst, 1967) or to intracellular release of the highly toxic phosphoramide mustard and acrolein from the primary metabolite specifically in tumour cells, but not in normal cells, which are probably able to break down the primary metabolite into non-toxic derivatives of cyclophosphamide (Connors *et al.*, 1974). The generalized toxicity of cyclophosphamide may be related to spontaneous breakdown of the primary metabolite in extracellular fluid (Connors *et al.*, 1974). Thus, it would appear that the cytotoxicity of cyclophosphamide to normal cells at any given concentration will depend upon their sensitivity or upon the permeability of their walls to the toxic breakdown products in the extracellular fluid. Moreover, the fact that the differentiated and stable odontogenic cells were apparently unaffected confirms that the cytotoxicity of cyclophosphamide may, in some way, be related to the process of cell division. The relative immunity of the odontogenic epithelium compared with the odontogenic mesenchyme to cytotoxic injury in the 40 mg group may thus be due to the difference in their mitotic indices (Chiba, 1965), to a difference in their permeability, biochemistry and biochemical activity or to all these factors. The evidence of some cytotoxic injury to the odontogenic epithelium and the increasing injury to the proliferating mesenchymal cells in the 80 mg and 120 mg groups may be related to the fact that, in the rat, the concentration and duration of cyclophosphamide in the tissues are directly related to the dose (Friedman, 1967; Brock *et al.*, 1971). Thus, increasing dosage not only appears to affect increasing numbers of susceptible cells in any one group (Rall, 1967) but also a wider variety of cell populations (Stekar, 1973).

That the undifferentiated mesenchymal cells in the proliferating zone of the pulp may be more sensitive than those of the odontogenic epithelium to the cytotoxicity of cyclophosphamide is suggested also by the changes in the pulp and the disruption in root growth seen 4 and 8 days after the injection of cyclophosphamide. Root growth in the rat incisor involves dentinogenesis as well as amelogenesis, and the present study has demonstrated that the development of both these structures was arrested in all experimental animals when examined 4 days after the injection of cyclophosphamide. The odontoblasts and other cells of the pulp are derived by division and differentiation of the undifferentiated mesenchymal cells in the proliferating zone of the pulp close to the basal odontogenic epithelium (Robins, 1967). Thus, injury to the undifferentiated mesenchymal cells may account for the arrest of dentinogenesis and for the acellularity of the pulp below the level of the last formed dentine. On the other hand, the proliferation of the mesenchymal cells appears to be dependent upon the inductive influence of the basal odontogenic epithelium (Tonge, 1967; Miller, 1969). Studies on Burkitt's lymphoma have suggested that root growth stops in those teeth in which, for some reason, the odontogenic epithelium is destroyed (Adatia, 1970, 1973). Therefore, it would appear that repopulation of the basal acellular area in the pulp with apparently normal pulp cells, the resumption of dentinogenesis and amelogenesis in the 40 mg group, and re-establishment of apparently normal basal odontogenic tissues in the 80 mg group by the eighth day suggest that the odontogenic epithelium had remained viable following the injection of cyclophosphamide or had recovered its functional potential.

Although abnormality in the formation of enamel might be suspected in the 80 mg and 120 mg groups, which showed evidence of some cytotoxic injury to the cells in the enamel organ, a further functional interdependence of the odontogenic epithelium and mesenchyme can explain the temporary cessation of amelogenesis even in the 40 mg group. Normally the epithelial cells differentiate into ameloblasts only after the cells proliferating from the odontogenic epithelium and migrating along the internal enamel epithelial layer come into contact with early dentine (Marsland, 1951). Consequently, cytotoxic injury limited to the mesenchymal cells may prevent the differentiation of ameloblasts as long as there is an absence of odontoblasts and of the inductive influence of their activity upon the cells of the internal enamel epithelial layer. Thus, the greater delay in the resumption of root growth with increasing dosage may be related to the longer time which might be required to repair the greater injury to the odontogenic mesenchyme with increasing dosage, to the injury to parts of odentogenic epithelium seen in the 80 mg and 120 mg groups, or to both these factors. The odontogenic aspects of this study have been discussed more fully elsewhere (Adatia, 1975).

Studies in rats examined 2 or more weeks after a single injection of upwards of 20 mg cyclophosphamide/kg have shown either poor development of teeth generally (Stekar, 1973) or dentinal hypoplasia in particular (Koppang, 1973). The present study appears to be the first to offer direct evidence to suggest that among the odontogenic cells in the incisor of the rat the cells of the undifferentiated mesenchyme in the proliferating zone of the pulp are most sensitive to the effects of cyclophosphamide. It appears also that the cytotoxic effects of 40 mg cyclophosphamide/kg on the odontogenic cells in the rat incisor may be localized to these undifferentiated mesenchymal cells. Thus, the observations reported in the present study could account for the lack of gross dental abnormality after a dose of 20 mg/kg or poor development of teeth after giving higher doses to rats up to 4 days old (Stekar, 1973), and for the microscopically demonstrable dentinal hypoplasia observed in older rats 2 weeks after injection of 25 mg–40 mg cyclophosphamide/kg (Koppang, 1973). It is, however, possible that the relative immunity of the odontogenic epithelium in the 40 mg group in the present study was only apparent and not real, for the injury might have been such that it could not be detected with the methods employed. Secondly, the cellular injury could have been such as to be repaired before mitosis. Thirdly, the damage could have been such as to need an added insult for the injury to become manifest. Nevertheless, if future work confirms the direct evidence of localized injury, as shown in the present study, it is clear that the incisor of the rat may provide, for two reasons, a valuable tool for the detailed investigation of cyclophosphamide *in vivo*. Firstly, the incisor of the rat appears to have a predictable pool of rapidly proliferating susceptible and more resistant cells in an accessible region throughout the life of the animal. Secondly, being normal cells, they are probably more likely to have predictable properties than malignant cells grown in tissue culture. Such work may, in turn, contribute to an understanding of the variable clinical response to cyclophosphamide observed in chemotherapy of cancer.

I wish to thank Mr D. Coles and Mrs J. Stephen for photographic and laboratory assistance.

REFERENCES

Adatia, A. K. (1968) Response of the Dental Elements to Chemotherapy of Burkitt's Tumour. *Int. dent. J.*, **18**, 646.

Adatia, A. K. (1970) Dental Aspects. In *Burkitt's Lymphoma*. Eds D. P. Burkitt and D. H. Wright. Edinburgh and London: Livingstone.

Adatia, A. K. (1973) Dental Changes in Burkitt's Lymphoma. *Path. Microbiol.*, **39**, 196.

Adatia, A. K. (1975) The Effects of Cyclophosphamide on Odontogenesis in the Rat. *Archs oral Biol.*, **20**, 141.

BROCK, N., GROSS, R., HOHORST, H.-J., KLEIN, H. O. & SCHNEIDER, B. (1971) Activation of Cyclophosphamide in Man and Animals. *Cancer, N.Y.*, **27**, 1512.

BROCK, N. & HOHORST, H.-J. (1967) Metabolism of Cyclophosphamide. *Cancer, N.Y.*, **20**, 900.

BURKITT, D. P., HUTT, M. S. R. & WRIGHT, D. H. (1965) The African Lymphoma: Preliminary Observations on Response to Therapy. *Cancer, N.Y.*, **18**, 399.

CALABRESI, P. & PARKS, R. E. JR (1970) Alkylating Agents, Antimetabolites, Hormones, and Other Antiproliferative Agents. In *The Pharmacological Basis of Therapeutics.* 4th edn. Eds L. S. Goodman and A. Gilman. New York: Macmillan.

CHIBA, M. (1965) Cellular Proliferation in the Tooth Germ of the Rat Incisor. *Archs oral Biol.*, **10**, 707.

CLIFFORD, P. (1966) Further Studies in the Treatment of Burkitt's Lymphoma. *E. Afr. med. J.*, **43**, 179.

CONNORS, T. A., COX, P. J., FARMER, P. B., FOSTER, A. B. & JARMAN, M. (1974) Some Studies of the Active Intermediates Formed in the Microsomal Metabolism of Cyclophosphamide and Isophosphamide. *Biochem. Pharmac.*, **23**, 115.

COOPER, E. H., FRANK, G. L. & WRIGHT, G. H. (1966) Cell Proliferation in Burkitt Tumour. *Eur. J. Cancer*, **2**, 377.

EPSTEIN, M. A. (1970) Long-term Tissue Culture of Burkitt's Lymphoma Cells. In *Burkitt's Lymphoma.* Eds. D. P. Burkitt and D. H. Wright. Edinburgh and London: Livingstone.

FRIEDMAN, O. M. (1967) Recent Biologic and Chemical Studies of Cyclophosphamide (NSC-26271). *Cancer chemother. Rep.*, **51**, 327.

GUARINO, A. M. & LITTERST, C. L. (1974) Metabolism of Cancer Chemotherapeutic Agents *via* Pathways Utilized by Xenobiotics. In *Antineoplastic and Immunosuppressive Agents*, Part I. Eds A. C. Sartorelli and D. G. Johns. Berlin, Heidelberg and New York: Springer-Verlag.

KOPPANG, H. S. (1973) Autoradiographic Investigations on the Effect of Cyclophosphamide on Dentinogenesis of the Rat Incisor. *Scand. J. dent. Res.*, **81**, 397.

LANE, M. (1967) Animal Investigations with Cyclophosphamide (NSC-26271): A Brief Survey. *Cancer chemother. Rep.*, **51**, 359.

MADOC-JONES, H. & MAURO, F. (1974) Site of Action of Cytotoxic Agents in the Cell Life Cycle. In *Antineoplastic and Immunosuppressive Agents*, Part I. Eds A. C. Sartorelli and D. G. Johns. Berlin, Heidelberg and New York: Springer-Verlag.

MARSLAND, E. A. (1951) A Histological Investigation of Amelogenesis in Rats—I: Matrix Formation. *Br. dent. J.*, **91**, 251.

MILLER, W. A. (1969) Inductive Changes in Early Tooth Development: I. A Study of Mouse Tooth Development on the Chick Chorioallantois. *J. dent. Res.*, **48**, 719.

RALL, D. P. (1967) Pharmacologic Aspects of Selective Chemotherapy of Leukemia and Burkitt's Tumor. Combination Chemotherapy: Advertent and Inadvertent. *Cancer Res.*, **27**, Pt I, 2650.

ROBINS, M. W. (1967) The Proliferation of Pulp Cells in Rat Incisors. *Archs oral Biol.*, **12**, 487.

STEKAR, J. (1973) Teratogenicity of Cyclophosphamides in Newborn Rats. *ArzneimForsch.*, **23**, 922.

TONGE, C. H. (1967) Identification of Cell Patterns in Human Tooth Differentiation. *J. dent. Res.*, **46**, 876.